# Educational Inequalities in the Transition to Adulthood in Belgium: The Impact of Intergenerational Mobility on Young-Adult Mortality in 2001-2009

**DOI:** 10.1371/journal.pone.0142104

**Published:** 2015-12-14

**Authors:** Hannelore De Grande, Hadewijch Vandenheede, Patrick Deboosere

**Affiliations:** Interface Demography, Department of Sociology, Faculty Economic and Social Sciences and Business Solvay School, Vrije Universiteit Brussel, Belgium, Pleinlaan 5, 1050 Brussels (Elsene), Belgium; Hunter College, UNITED STATES

## Abstract

Several studies have focused on the association between parental and personal socioeconomic position (SEP) and health, with mixed results depending on the specific health outcome, research methodology and population under study. In the last decades, a growing interest is given to the influence of intergenerational mobility on several health outcomes at young ages. This study addresses the following research question: Is educational intergenerational mobility associated with all-cause and cause-specific mortality in young adulthood? To this end, the Belgian 1991 and 2001 censuses are used, providing characteristics of young persons at two time points (T1 = 01/03/91;T2 = 01/10/01) and follow-up information on mortality and emigration between T2 and 31/12/09 (T3). The study population consists of all official inhabitants of Flanders and the Brussels-Capital Region at T2, born between 1972 and 1982 and alive at T2. Parental and personal education are divided into primary (PE), lower secondary (LSE), higher secondary (HSE) and higher education (HE). We analyse mortality between T2 and T3 calculating age-standardised mortality rates (ASMRs) and using Cox regression (hazard ratios = HR). Personal rather than parental education determines the observed mortality rates, with high all-cause mortality rates among those with PE, irrespective of parental education (e.g., among men ASMR_PE-PE_ = 200.0 [95% CI 158.0–241.9]; ASMR_HE-PE_ = 319.7 [183.2–456.3]) and low all-cause mortality among those in higher education, regardless of parental education (ASMR_PE-HE_ = 41.7 [30.8–52.6]; ASMR_HE-HE_ = 38.0 [33.2–42.8]). There is some variation by gender and according to cause of death. This study shows the strong association between personal education and young-adult mortality.

## Background

To what extent is the social background of your parents or your own socioeconomic position (SEP) most important in determining health outcomes in young adulthood? Numerous studies have focused on the association between parental and personal SEP and health, with mixed results depending on the specific health outcome, research methodology and population under study [1–4]. In the last decades, a growing interest is given to the influence of intergenerational mobility on several health outcomes at young ages [5–8]. Research of Deboosere, Gadeyne, Charafeddine & Van Oyen [9] found an impact for both parental and personal education on self-rated health in Belgium, but with a more profound association of the latter. This paper deepens this earlier study through exploring to what extent an association does exist between intergenerational educational mobility and *young-adult mortality* in Belgium, which is the main research question of this study.

### Education as a driver of mobility between generations?

Education is an important indicator for SEP, especially in young adulthood. Several authors have demonstrated that education is strongly linked to different health outcomes in young adulthood [6,10,11] and that education affects other measures of SEP later in life such as income and occupation [12–14]. For most young adults their educational level is finalised at age 25 and not altered later in life, in contrast to other SEP measures that show variation over the life course until retirement [15]. Income and occupation are less relevant determinants at a young age, as these often not yet reflect the status young adults have obtained. Education has qualities that go beyond those of other resources because it builds resourcefulness, or the ability to meet situations effectively, creating skills and abilities called human capital [16]. Education is considered the most relevant indicator for SEP at a young age [17] and will thus be the focus of this study.

The educational level one achieves in society is strongly determined by one’s social origin [18]. Parental occupation, status and education all have independent effects on educational attainment of the child, with parental education of the greatest and increasing importance [19]. Educational mobility has made some impressive jumps over the last generations, largely due to the democratisation of education [20]. This is similar for most welfare states: young adults are generally better educated than their parents. This upward mobility trend is the result of two mechanisms: educational policies aiming at providing education for all, and the need for further specialisation and demand for a high-educated workforce in the labour market [21,22]. While intergenerational mobility in education is high, the composition of the society according to other measures of SEP does not necessarily change at the same pace. Education can be an important ticket to a better position, but educational mobility is not equal to social mobility [21], as this is also related to other factors making up one’s social class.

Does this shift in educational achievement also influence the health risks of young adults? In the next section, the links between intergenerational mobility and health will be further detailed.

### Intergenerational mobility & health

The links between education and health are firmly established [23–26]. Both education of the parents and personal educational level are important in this respect [10,16,27]. Many studies have focused on the beneficial effects of upward social mobility on health, as well as on the negative consequences of downward social mobility [1,2,28]. Some studies suggest that adversities in childhood have a persistent impact on health over the life course [29,30], implying that health returns on education are smaller for those starting from a disadvantaged position, as these adversities cannot be cancelled out by adding other resources [31]. Those that benefit from a protective start in life are better equipped to reap the educational rewards [32]. This is also referred to as “*cumulative advantage*” [33] or “*resource multiplication*” [10,31]. Applied to intergenerational mobility, the health of upwardly mobile persons should hence be better than the health of the class of origin, but still worse than the health of the class of destination, as some of the behaviours of the class of origin are somewhat ingrained in the behaviour of the mobile ones. Continuing to draw on the same logic, downwardly mobile persons will still carry the assets of the higher strata, lending them better health prospects than the stable low classes.

Other studies suggest that upward mobility increases healthy behaviour, while downward mobility enhances problematic or risky behaviour, as the educationally mobile group copies the behaviour of the stable group they end up in [34], making the destination position most influential to different health outcomes or composite health measures [35,36]. This is in part related to the *resource substitution theory* [16], stating that the beneficial health effects are largest among persons starting with fewer alternatives, e.g. with poorly educated parents, as education can overcome these adversities in health, and give them similar opportunities as those growing up in well-off families. Rather than the mobility pattern itself, the destination position is most decisive in these studies.

Considering downward mobility, health selection processes need to be accounted for as well. Poor health in childhood can hamper the educational trajectory, and long-term illnesses can irreversibly break off the educational path, leaving the young adult with poor prospects both in terms of SEP and health [27]. There is little doubt that health can lead to downward mobility, but debate continues on how strong this relation is [2,37].

### Mortality as ultimate health indicator

Mortality is a universal indicator for population health and can also be used as an indicator of economic success or failure on the societal level [38]. In young adulthood, most mortality is preventable and a high cost to society [39]. Analysing cause-specific mortality can give insight into the social patterning of risk behaviours and mental health problems. This is particularly so for preventable causes such as suicides, road accidents and violence, which are among the main causes of death at a young age in Western societies [40]. In contrast to health outcomes such as health behaviour or self-assessed health, only few studies have looked into the effects of intergenerational mobility on cause-specific mortality at a young age [7,41,42] compared to into older age [5,43], and even fewer specifically look into educational mobility. Most use parental social class rather than parental education to assess social mobility, except for Remes & Martikainen on young adults leaving the parental home and Elo, Martikainen, & Myrskylä [44] among older adults. Both studies found significant mediating of childhood conditions by SEP in (young) adulthood on several causes of death. There is need for more research using large datasets in order to find robust results [45].

Therefore census data, including information on the total young-adult population in Belgium, are used. In this paper the following research question will be put to the test: To what extent does an association between intergenerational educational mobility and young-adult mortality exist? This research question is further divided into the following sub questions:

1. To what extent are inequalities in young-adult mortality associated with both personal and parental educational level?
2. To what extent is the association found in (1) similar for all main causes of death?
3. Does the association between educational mobility and mortality hold after adjusting for other SEP measures (i.e. housing and employment situation) in childhood and in young adulthood, and health measured in young adulthood?

## Methods & Material

### Data

Data are derived from the Belgian 1991 and 2001 censuses, providing characteristics of young persons at two time points (T1 = 01/03/91;T2 = 01/10/01) and follow-up information on mortality and emigration between T2 and 01/01/10 (T3). Belgian census data were collected and prepared by Statistics Belgium and include all persons living de jure in Belgium. The final database is composed of several data sources. In a first stage, the census data were linked to the national registry, providing information on migration and all-cause mortality data. In a second stage, death certificates were added, with information on causes of death. For this study, a third linkage combined information of both censuses into one database. Statistics Belgium made an identification key to obtain a 100% linkage between the 1991 and 2001 census for those persons living in Belgium both at T1 and T2. No ethical approval was obtained, because it was not applicable to our data. These data were anonymized before delivery and analysis and were approved for statistical analysis restricted to our research unit (Interface Demography) by the Belgian Privacy Commission.

The study population consists of all official inhabitants of Flanders and the Brussels-Capital Region at T2, born between 1972 and 1982 (N = 880,107). These birth cohorts were delineated for two reasons. First, the age of these persons at the 1991 census ranges between 8–19 years: more than 95% of these children/adolescents still live at home, which is a condition sine qua non for measuring parental education (see childhood characteristics). Second, at the 2001 census, the age ranges between 19 and 29 years. One in five is still studying, meaning that their final completed educational level can vary from their current situation. A young adult older than eighteen still in secondary education however clearly indicates trailing behind the educational track. We assume that the current educational level is a good indicator of the future SEP of young adults, conform to an earlier study where the lower age limit was confined to 19 year-olds as well [46]. Using only completed education showed a rather strong underestimation of upward mobility and an overestimation of those having the same educational level as their parents. However, estimates of the Cox regression were only slightly affected, and the conclusions of the results are similar when using completed or current educational level.

### Childhood characteristics

The distribution of each variable measuring childhood characteristics at T1 and young-adult characteristics at T2 is presented in Table 1 separately for men and women.

**Table 1:** Overview of childhood and young-adult characteristics at T2 (01/10/2001): numbers (N) and percentages (%).

	men		women	
	N	%	N	%
Total	448,996		431,111	
*Childhood characteristics*				
**Parental education**				
LE	108,559	25.4	104,066	25.3
LSE	74,154	17.3	70,451	17.2
HSE	118,857	27.8	113,700	27.7
HE	117,667	27.5	112,124	27.3
not living with parents	8,611	2.0	10,482	2.5
missing	21,148	4.7	20,288	4.7
**parental employment status**				
self-employed	71,653	16.6	67,133	16.3
white collar employee	159,119	36.9	152,83	37.1
blue collar employee	138,100	32.0	131,247	31.9
not employed	62,495	14.5	60,558	14.7
missing	17,629	3.9	19,343	4.5
**parental housing status**				
owner & high to medium comfort	239,233	55.1	227,861	54.8
tenant & high to medium comfort	57,372	13.2	56,684	13.6
owner & low to no comfort	92,497	21.3	87,809	21.1
tenant & low to no comfort	44,896	10.4	43,736	10.5
missing	14,998	3.3	15,021	3.5
*Young-adult characteristics*				
**Personal education**				
LE	11,525	2.7	7,547	1.8
LSE	57,097	13.4	35,019	8.4
HSE	191,587	44.9	161,972	39.0
HE	166,720	39.0	211,110	50.8
missing	22,067	4.9	15,463	3.6
**personal employment status**				
self-employed	29,120	6.8	19,002	4.6
white collar employee	131,879	30.5	182,571	43.7
blue collar employee	102,457	23.7	33,114	7.9
job seeker	35,132	8.1	39,485	9.5
not employed, nor seeking a job	5,453	1.3	12,651	3.0
student	82,809	19.2	89,984	21.5
rest category	44,742	10.4	41,104	9.8
missing	17,404	3.9	13,200	3.1
**personal housing status**				
owner & high to medium comfort	203,191	49.4	189,865	47.8
tenant & high to medium comfort	84,424	20.5	73,825	18.6
owner & low to no comfort	79,357	19.3	86,565	21.8
tenant & low to no comfort	44,203	10.8	46,894	11.8
missing	37,821	8.4	33,962	7.9
**longstanding illness**				
no	374,256	91.0	366,863	91.4
yes	37,003	9.0	32,681	8.6
missing	37,737	8.4	31,567	7.3

*Parental education* is measured using the highest educational level obtained by one of the parents within the household. For one-parent households, the diploma of the single parent, being a father or a mother, within that household is used. Educational level of the father and mother were also included in the analyses separately, leading to similar results as using the highest educational level of one of the parents. As especially fathers’ education led to a high percentage of missings (most one-parent households are single mothers), the highest parental education was used rather than the fathers’ or mothers’ education. Parental education is coded into 4 groups according to the ISCED-classification: 1 maximum primary education (ISCED 0–1), 2 lower secondary education (ISCED 2), 3 higher secondary education (ISCED 3–4) and 4 higher education (ISCED 5+). Those not living with their parents at T1 will be included as a separate category in the analyses (2.0% of total study male population, 2.6% of the female population—see Table 1). Missing values did not exceed 5% of the total study population.

Other childhood characteristics could also contribute to educational differences in young-adult mortality. Childhood characteristics are mostly measured by occupational class of the father, but also by housing characteristics, home ownership, mother’s marital status, etc. [43]. Adult mortality risks have found to be higher among those exposed to poorer childhood circumstances [5,43]. Two other SEP variables will be included in the analysis. As a proxy for parental wealth the *housing status* of the household is used: this is a combination of tenure with comfort of the housing. There are four categories: 1 owner & high to medium comfort, 2 tenant & high to medium comfort, 3 owner & little to no comfort, 4 tenant & little to no comfort. The other indicator for SEP in the household is the *parental employment status*, i.e. the employment status of the head of the household. Normally, this is the status of the father, except for single-mother households. Four categories are used: 1 self-employed, 2 white-collar employee, 3 blue-collar employee, 4 not employed.

### Young-adult characteristics

For personal educational level the same classification as that of parental education is applied. The *current educational level enrolled in* is used for those still in education. The difference between highest education level completed and enrolled in lies largely in the % categorised in HSE and HE. The same analyses were run using only completed educational level and this did not change the interpretation of the results.

Analogous to the other SEP measures used in childhood, similar measures for housing and employment situation at T2 are used. *Personal employment situation* includes extra categories compared to the measure at T1, measured as 1 self-employed, 2 white-collar employee, 3 blue-collar employee, 4 job seeker, 5 not employed, nor seeking a job (including personal and familial reasons not to work, such as informal care giving and being on long-term sick leave), 6 student and 7 rest category.

There is no information on the health status of the children living with their parents at T1, but there is information on the health situation of the young adults at T2. Information on both subjective health and longstanding illness or impairment is available. The latter is included in the analyses as it is a better indicator for severe health problems than self-reported health. It is categorised as a dummy variable with 0 no longstanding illness or impairment and 1 longstanding illness or impairment.

### Outcome variables

The causes of death are coded into the International Classification of Diseases version 10 (ICD-10). The analyses are performed for the major causes of death in young adulthood: suicide, including undetermined causes of death (X60-X84,Y10-Y34); road accidents (V00-V89); other external and drug- and alcohol-related deaths (V90-Y98 excl. suicides and road accidents, F10-F16, F18-19, G31.2, G62.1 I42.6, K29.2, K70,K86.0); cancers (C00-D48) and other natural deaths due to medical conditions (A00-R99, excluding cancers and drug- and alcohol-related deaths).

### Methods

We analyse all-cause and cause-specific mortality between T2 and T3 using Cox regression with parental education, housing status and employment situation at T1; personal education, housing status and employment, as well as long-standing illness or impairment at T2. First of all, age-standardised mortality rates (ASMRs), using direct standardisation with the Belgian population in 2001 as standard, will be presented for all cross-classifications between parental and personal education (as presented in Table 1) for all-cause mortality. For each ASMR a 95% confidence interval (CI) is calculated as well. Censoring is used at date of death or out-migration to calculate the person years from T2 to T3. Second, hazard ratios (HR) are calculated using Cox regression to analyse both all-cause and cause-specific mortality between T2 and T3. The Cox proportional hazard assumption was formally tested through the Schoenfeld residual test in Stata and is not violated. All models are controlled for age. Missings on either parental or personal education are left out of the analysis included in S1 Appendix and S2 Appendix. All analyses are run with Stata MP 13.1.

## Results

### Educational mobility in Belgium

The transition matrix (Table 2) shows a cross-classification of parental and personal educational level. There is a general trend towards upward mobility, with few young adults with only primary education. Even among those with parents who only completed primary education, just 5.5% of the boys and 3.6% of the girls end up with only primary education. Among those with parents with higher education, less than 1% of the boys and girls obtain only primary education. The majority of the young adults finish at least higher secondary education. Two thirds of the boys of parents with higher education also end up in higher education, among girls this amounts to 80.1%. In the next sections the different mobility patterns will be referred to starting with the abbreviation of the parental education, followed by personal education (e.g. PE-HSE: young adult with primary-educated parents who completed higher-secondary education).

**Table 2:** Transition matrix of parental and personal educational level among young adults born between 1972 and 1982^1^, in numbers and row percentages, stratified by sex.

parental education	personal educational level				
**men**	**PE**	**LSE**	**HSE**	**HE**	**Total**
PE	5,478	23,335	54,304	17,023	100,140
	5.5	23.3	54.2	17.0	
LSE	1,907	11,565	38,331	18,428	70,231
	2.7	16.5	54.6	26.2	
HSE	2,014	12,320	55,455	44,569	114,358
	1.8	10.8	48.5	39.0	
HE	829	4,603	31,561	79,080	116,073
	0.7	4.0	27.2	68.1	
Total	10,228	51,823	179,651	159,100	400,802
**women**	**PE**	**LSE**	**HSE**	**HE**	**Total**
PE	3,590	16,388	53,645	24,889	98,512
	3.6	16.6	54.5	25.3	
LSE	1,093	6,216	33,019	27,145	67,473
	1.6	9.2	48.9	40.2	
HSE	1,131	5,913	43,455	59,893	110,392
	1.0	5.4	39.4	54.3	
HE	509	1,835	19,802	89,193	111,339
	0.5	1.6	17.8	80.1	
Total	6,323	30,352	149,921	201,120	387,716

Data: Census 1991–2001 linked to national register, own calculations

^1^: living in Flanders or the Brussels-Capital Region on 01/10/2001

Educational categories: primary education (PE), lower secondary education (LSE), higher secondary education (HSE), higher education (HE)

Missing values on either parental or personal education: 39,583 (9.1%) men; 32,913 (7.8%) women

### Educational mobility and all-cause mortality

The transition matrix in Table 3 depicts age-standardised mortality rates and number of deaths for each cross-classification stratified by sex. The table can be read from the viewpoint of parental education at T1 (column totals) or personal education at T2 (row totals) or a combination of both (cross-classified cells). Among both men and women, educational differences are more pronounced when differentiating between personal rather than parental educational levels. Among men from PE families 110.6 per 100,000 died between 2001–2009, compared to 59.1 from HE families, while the mortality rates of men with PE are more than fourfold the rate of men with HE (ASMR_own PE_ = 225.4 [95% CI 193.1–257.7] compared to ASMR_own HE_ = 40.6 [37.2–44.1]). Among women differences between parental educational levels are less pronounced than among men, with only significant differences between the lowest and highest educated (ASMR_parental PE_ = 37.7 [95% CI 33.5–42.0] compared to ASMR_parental HE_ = 27.9 [37.2–44.1]). Large and significant differences are found between each personal educational level, in the same order as among men.

**Table 3:** Age-standardised mortality rates per 100,000 person years, 95% confidence intervals between brackets, and number of deaths between 2001–2009 for each intersection between parental and personal education and totals, stratified by sex.

parental education	personal educational level				
**men**	**PE**	**LSE**	**HSE**	**HE**	**Total**
PE	200.0 [158.0–241.9]	142.2 [125.2–159.2]	109.4 [99.6–119.2]	41.7 [30.8–52.6]	110.6 [103.4–117.9]
	88	271	488	58	905
LSE	196.2 [127.2–265.2]	135.4 [111.9–158.9]	96.2 [85.4–107.0]	38.6 [28.7–48.6]	89.9 [82.2–97.6]
	31	128	305	59	523
HSE	297.9 [212.6–383.3]	171.2 [145.2–197.3]	89.6 [80.9–98.4]	45.5 [38.6–52.4]	84.4 [78.5–90.3]
	47	169	407	167	790
HE	319.7 [183.2–456.3]	129.7 [92.7–166.8]	95.1 [83.0–107.2]	38.0 [33.2–42.8]	59.1 [54.2–64.0]
	21	48	243	242	554
Total	225.4 [193.1–257.7]	146.1 [134.6–157.6]	98.3 [93.3–103.4]	40.6 [37.2–44.1]	
	187	616	1443	526	
**women**	**PE**	**LSE**	**HSE**	**HE**	**Total**
PE	63.5 [34.3–92.6]	56.4 [43.6–69.2]	38.0 [32.2–43.8]	20.8 [14.5–27.2]	37.7 [33.5–42.0]
	19	76	167	43	305
LSE	176.9 [90.2–263.5]	44.5 [26.3–62.7]	37.6 [30.3–44.8]	26.6 [19.9–33.3]	36.1 [31.2–41.1]
	16	23	103	61	203
HSE	176.4 [90.0–262.7]	69.2 [45.5–92.9]	37.1 [30.7–43.5]	23.4 [19.2–27.7]	32.7 [28.9–36.4]
	16	33	130	115	294
HE	199.7 [61.1–338.2]	65.5 [24.7–106.2]	41.9 [31.4–52.3]	23.3 [19.7–26.8]	27.9 [24.4–31.3]
	8	10	63	167	248
Total	115.9 [86.1–145.7]	57.1 [47.7–66.6]	37.7 [34.3–41.2]	23.6 [21.2–25.9]	
	59	142	463	386	

**Data:** Census 1991–2001 linked to national register, own calculations

**Study population**: born between 1972 and 1982 and living in Flanders or the Brussels-Capital Region on 01/10/2001

Educational categories: primary education (PE), lower secondary education (LSE), higher secondary education (HSE), higher education (HE)

ASMR_men_ children not living with parents at T1: 125.8 [98.4–153.3]

ASMR_men_ Missing education: 111.2 [97.1–125.3]

ASMR_women_ children not living with parents at T1: 45.8 [30.3–61.4]

ASMR_women_ Missing education: 58.1 [47.3–68.8]

This pattern is recurrent in the cross-classified cells. Upwardly mobile men from LSE families who are HE have similar mortality rates as those who are stable HE (both parental and personal education is high; ASMR_LSE-HE_ = 38.6 [28.7–48.6]; ASMR_HE-HE_ = 38.0 [33.2–42.8]). Downwardly mobile men from HE towards LSE have similar rates as the stable lower secondary educated (ASMR_HE-LSE_ = 129.7 [92.7–166.8]; ASMR_LSE-LSE_ = 135.4 [111.9–158.9]). In general, a similar pattern appears among women. Mortality rates within each personal educational level differ most among PE women, but confidence intervals are wide because of few deaths per cross-classified group (for example: ASMR_PE-PE_ = 63.5 [34.3–92.6];ASMR_PE-HE_ = 199.7 [61.1–338.2]).

**Fig 1:**
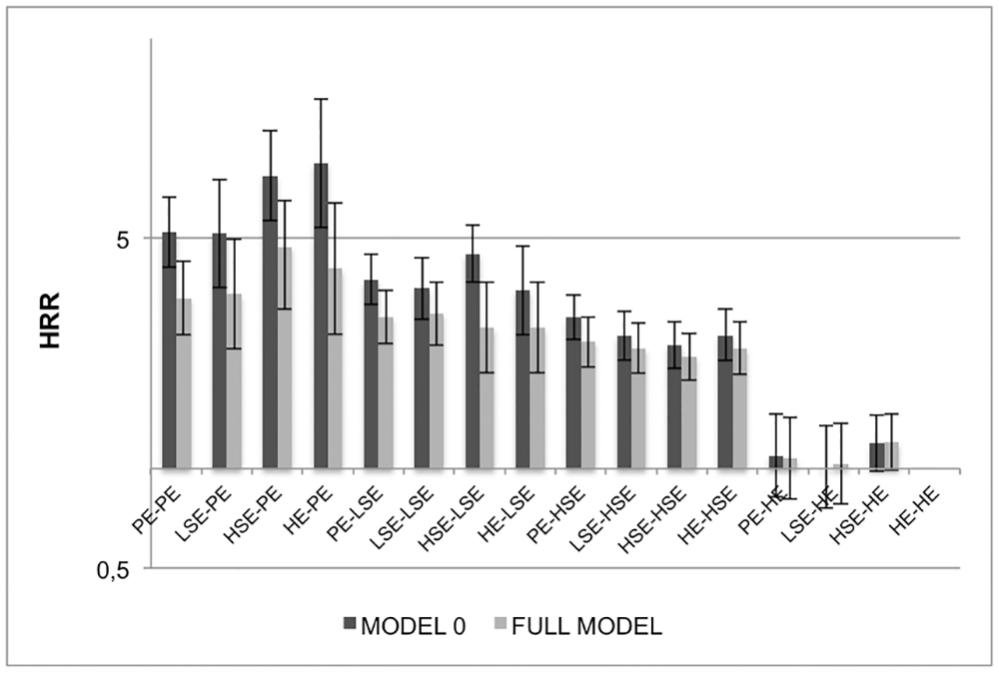
PE = primary education, LSE = lower secondary education, HSE = higher secondary education, HE = higher education. Model 0: controlled for age; Full model including all controls: parental and personal housing comfort, personal employment situation, long-term illness/disability, age.

**Fig 2:**
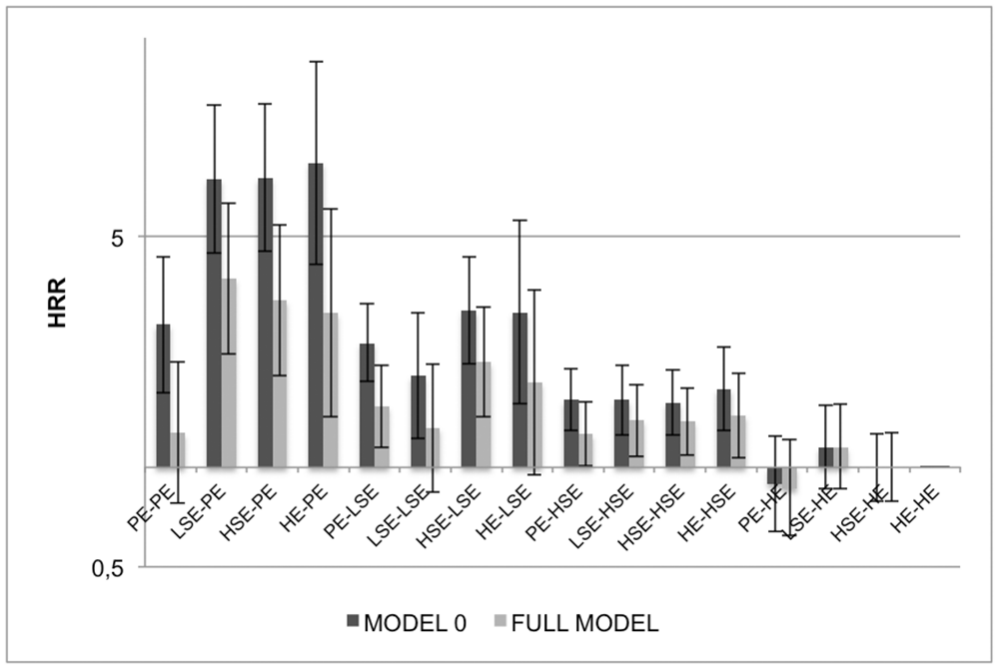
PE = primary education, LSE = lower secondary education, HSE = higher secondary education, HE = higher education. Model 0: controlled for age; Full model including all controls: personal housing comfort, personal employment situation, long-term illness/disability, age.

These results are reflected in the Cox regression analysis in Table 4 for men and Table 5 for women. Model 1 includes the independent associations of parental and personal education, adjusted for age, and model 2 includes parental and personal education mutually adjusted. Table 4, Model 1 shows a strong gradient in mortality by personal education, and small but significant mortality differences according to parental education. After including both variables in one model (model 2), none of the parental educational differences remain significant, except for men not living with their parents in 1991 (HR_model2_ = 1.28 [1.01–1.62]). Personal educational levels are only slightly affected through controlling for parental education. Among women, the association between parental education and young-adult mortality reverses in model 2: while women from PE families had a 36% higher mortality rate than those from HE families in model 1, the mortality rate becomes lower than in HE families after controlling for personal education (HR_parental PE model2_ = 0.87 [0.73–1.05]). Controlling for parental education leads to slight increases in the hazard ratios of personal education. This will be elaborated in Figs 1 and 2 in the last part of the results section.

**Table 4:** Hazard ratios and 95% confidence intervals for all-cause and cause-specific mortality between 2001–2009 according to parental and personal education among young-adult MEN.

	**ALL-CAUSE MORTALITY**		**CANCERS** ^A^ **(N**_**d**_ **= 262)**		**OTHER DISEASES (excl. Cancer) (N**_**d**_ **= 402)**	
	model 1	model 2	model 1	model 2	model 1	model 2
**Parental Education**						
PE	1.86 [1.68–2.06]***	1.08 [0.98–1.21]	1.41 [1.00–1.99]*	1.12 [0.77–1.64]	2.02 [1.51–2.70]***	0.99 [0.73–1.35]
LSE	1.51 [1.34–1.70]***	1.00 [0.88–1.13]	1.02 [0.68–1.54]	0.88 [0.58–1.36]	1.46 [1.05–2.05]*	0.91 [0.64–1.28]
HSE	1.42 [1.27–1.58]***	1.07 [0.95–1.19]	1.29 [0.92–1.83]	1.19 [0.83–1.70]	1.55 [1.15–2.09]**	1.13 [0.83–1.53]
HE	1.00	1.00	1.00	1.00	1.00	1.00
not living with parents in 1991	2.14 [1.70–2.70]***	1.28 [1.01–1.62]*	0.96 [0.35–2.65]	0.76 [0.27–2.11]	1.61 [0.77–3.35]	0.78 [0.37–1.64]
missing	1.37 [1.13–1.66]**	0.88 [0.72–1.07]	1.13 [0.60–2.16]	0.94 [0.48–1.81]	1.36 [0.79–2.34]	0.72 [0.42–1.26]
**Personal education**						
PE	5.31 [4.52–6.23]***	5.18 [4.38–6.11]***	3.46 [2.07–5.77]***	3.46 [2.07–5.77]***	11.1 [7.6–16.2]***	11.6 [7.8–17.2]***
LSE	3.51 [3.14–3.93]***	3.44 [3.05–3.88]***	1.73 [1.18–2.52]**	1.72 [1.18–2.52]**	3.29 [2.34–4.63]***	3.39 [2.36–4.87]***
HSE	2.39 [2.17–2.64]***	2.35 [2.13–2.61]***	1.11 [0.82–1.50]	1.12 [0.82–1.51]	2.31 [1.72–3.09]***	2.34 [1.72–3.17]***
HE	1.00	1.00	1.00	1.00	1.00	1.00
missing	4.62 [3.81–5.60]***	4.53 [3.72–5.51]***	1.23 [0.49–3.06]	1.23 [0.49–3.06]	18.6 [13.0–26.7]***	19.3 [13.3–28.1]***
**EXTERNAL DEATHS**	**SUICIDES** ^A^ ^**,**^ ^B^ **(N**_**d**_ **= 950)**		**ROAD ACCIDENTS (N**_**d**_ **= 887)**		**OTHER VIOLENT DEATHS** ^C^ **(N**_**d**_ **= 381)**	
	model 1	model 2	model 1	model 2	model 1	model 2
**Parental Education**						
PE	1.82 [1.51–2.20]***	1.04 [0.85–1.27]	1.99 [1.62–2.44]***	1.18 [0.95–1.47]	3.19 [2.30–4.43]***	1.48 [1.04–2.09]*
LSE	1.42 [1.15–1.76]**	0.92 [0.74–1.15]	1.98 [1.59–2.47]***	1.29 [1.03–1.62]*	2.33 [1.61–3.36]***	1.26 [0.87–1.84]
HSE	1.31 [1.08–1.59]**	0.97 [0.79–1.18]	1.74 [1.42–2.13]***	1.28 [1.04–1.62]*	1.94 [1.37–2.74]***	1.25 [0.88–1.79]
HE	1.00	1.00	1.00	1.00	1.00	1.00
not living with parents in 1991	2.84 [1.97–4.08]***	1.67 [1.15–2.42]**	2.32 [1.48–3.63]***	1.45 [0.92–2.28]	3.01 [1.52–5.96]**	1.44 [0.72–2.88]
missing	1.46 [1.05–2.03]*	0.93 [0.66–1.30]	1.18 [0.79–1.78]**	0.79 [0.53–1.19]	2.26 [1.32–3.89]**	1.20 [0.69–2.08]
**Personal education**						
PE	5.48 [4.10–7.32]***	5.31 [3.93–7.18]***	3.03 [2.04–4.48]***	2.87 [1.92–4.28]***	12.8 [8.0–20.6]***	11.0 [6.7–18.1]***
LSE	3.91 [3.20–4.79]***	3.84 [3.10–4.77]***	3.94 [3.18–4.89]***	3.71 [2.95–4.66]***	8.01 [5.47–11.7]***	6.99 [4.68–10.4]***
HSE	2.48 [2.08–2.96]***	2.47 [2.05–2.97]***	2.95 [2.45–3.55]***	2.78 [2.29–3.37]***	4.81 [3.37–6.86]***	4.36 [3.02–6.29]***
HE	1.00	1.00	1.00	1.00	1.00	1.00
missing	2.81 [1.82–4.34]***	2.74 [1.76–4.26]***	3.05 [1.95–4.78]***	2.92 [1.85–4.60]***	7.88 [4.25–14.61]***	6.91 [3.69–13.0]***

**Study population**: born between 1972 and 1982 and living in Flanders or the Brussels-Capital Region on 01/10/2001

Model 1: independent effects and controlled for age, Model 2: mutually adjusted and controlled for age

*: p<0.050;

**: p<0.010;

***: p<0.001

^A^ = inclusive of drug- or alcohol related deaths, excluding suicides and road accidents;

^B^: inclusive of undetermined deaths;

^C^ = inclusive of associated and contributing causes of death

**Table 5:** Hazard ratios and 95% confidence intervals for all-cause and cause-specific mortality between 2001–2009 according to parental and personal education among WOMEN.

	**ALL-CAUSE MORTALITY**		**CANCERS** ^A^ **(N**_**d**_ **= 298)**		**OTHER DISEASES (excl. Cancer) (N**_**d**_ **= 288)**	
	model 1	model 2	model 1	model 2	model 1	model 2
**Parental education**						
PE	1.36 [1.16–1.61]***	0.87 [0.73–1.05]	1.13 [0.81–1.56]	0.91 [0.64–1.31]	1.97 [1.40–2.79]***	0.80 [0.55–1.16]
LSE	1.31 [1.09–1.57]**	0.99 [0.82–1.19]	1.15 [0.80–1.65]	1.00 [0.68–1.45]	1.44 [0.96–2.14]	0.80 [0.52–1.21]
HSE	1.17 [0.99–1.38]	0.98[0.82–1.16]	1.17 [0.85–1.62]	1.07 [0.77–1.49]	1.37 [0.95–1.97]	0.94 [0.65–1.36]
HE	1.00	1.00	1.00	1.00	1.00	1.00
not living with parents in 1991	1.60 [1.14–2.25]**	0.92 [0.65–1.31]	0.65 [0.26–1.61]	0.50 [0.20–1.27]	2.51 [1.33–4.73]**	0.80 [0.41–1.54]
missing	1.44 [1.09–1.89]	0.98 [0.74–1.31]	1.15 [0.65–2.05]	0.97 [0.54–1.75]	1.98 [1.14–3.43]*	0.87 [0.50–1.54]
**Personal education**						
PE	4.78 [3.71–6.15]***	5.03 [3.87–6.54]***	1.87 [0.95–3.67]	2.05 [1.03–4.09]*	18.3 [12.1–27.8]***	20.2 [13.0–31.5]***
LSE	2.45 [2.04–2.93]***	2.57 [2.12–3.12]***	1.26 [0.83–1.90]	1.35 [0.87–2.10]	5.93 [4.08–8.63]***	6.56 [4.39–9.82]***
HSE	1.60 [1.40–1.82]***	1.65 [1.43–1.90]***	1.33 [1.04–1.70]	1.37 [1.05–1.79]*	2.57 [1.86–3.55]***	2.75 [1.96–3.88]***
HE	1.00	1.00	1.00	1.00	1.00	1.00
missing	5.29 [4.03–6.95]***	5.51 [4.17–7.28]***	2.93 [1.54–5.58]***	3.13 [1.63–6.02]***	21.1 [13.7–32.7]***	22.9 [14.6–36.0]***
**EXTERNAL DEATHS**	**SUICIDES** ^A^ ^**,**^ ^B^ **(N**_**d**_ **= 264)**		**ROAD ACCIDENTS (N**_**d**_ **= 179)**		**OTHER VIOLENT DEATHS** ^C^ **(N**_**d**_ **= 98)**	
	model 1	model 2	model 1	model 2	model 1	model 2
**Parental education**						
PE	0.89 [0.63–1.26]	0.59 [0.40–0.85]**	1.64 [1.07–2.50]*	1.30 [0.82–2.07]	4.27 [2.05–8.89]***	2.31 [1.05–5.06]*
LSE	1.11 [0.77–1.60]	0.83 [0.57–1.21]	1.74 [1.11–2.73]*	1.49 [0.93–2.38]	3.36 [1.52–7.43]**	2.27 [1.00–5.17]
HSE	0.87 [0.62–1.22]	0.72 [0.51–1.03]	1.15 [0.74–1.78]	1.04 [0.66–1.62]	2.95 [1.39–6.27]**	2.33 [1.08–5.02]*
HE	1.00	1.00	1.00	1.00	1.00	1.00
not living with parents in 1991	1.61 [0.85–3.06]	0.99 [0.51–1.94]	1.06 [0.33–3.44]***	0.83 [0.25–2.74]	6.31 [2.10–18.95]**	2.98 [0.95–9.37]
missing	0.99 [0.54–1.83]	0.70 [0.38–1.32]	0.83 [0.33–2.11]	0.70 [0.25–2.74]	1.98 [0.54–7.32]	1.17 [0.95–9.37]
**Personal education**						
PE	2.54 [1.28–5.03]**	2.97 [1.47–6.00]**	2.56 [1.11–5.91]*	2.36 [1.01–5.54]*	10.5 [4.9–22.6]***	8.33 [3.74–18.6]***
LSE	2.34 [1.61–3.41]***	2.78 [1.85–4.17]***	1.69 [1.02–2.81]*	1.54 [0.90–2.63]	5.7 [3.3–10.2]***	4.56 [2.47–8.42]***
HSE	1.56 [1.19–2.05]**	1.78 [1.33–2.38]***	1.53 [1.11–2.10]*	1.40 [0.99–1.98]	2.11 [1.27–3.50]**	1.73 [1.01–2.94]**
HE	1.00	1.00	1.00	1.00	1.00	1.00
missing	1.89 [0.77–4.65]	2.17 [0.87–5.37]	2.08 [0.76–5.71]	1.95 [0.70–5.40]	4.54 [1.37–15.08]*	3.75 [1.11–12.7]*

**Study population**: born between 1972 and 1982 and living in Flanders or the Brussels-Capital Region on 01/10/2001

Model 1: independent effects and controlled for age, Model 2: mutually adjusted and controlled for age

*: p<0.050;

**: p<0.010;

***: p<0.001

Educational categories: primary education (PE), lower secondary education (LSE), higher secondary education (HSE), higher education (HE)

^A^ = inclusive of associated and contributing causes of death;

^B^: inclusive of undetermined deaths;

^C^ = inclusive of drug- or alcohol related deaths, excluding suicides

### Educational mobility and cause-specific mortality

To answer the second research question concerning to what extent parental and personal education are similarly associated with each cause of death group as with all-cause mortality, Tables 4 and 5 also show HR for the most common causes of death. Overall, there are smaller inequalities in parental compared to personal education (Model 1). For most causes of death, differences in parental education disappear after inclusion of personal education (Model 2). The relation between personal education and different causes of death is little influenced by parental education.

Among men, parental education is still significant in Model 2 concerning road accidents and other external death except suicide. There is also a higher HR for suicide among men not living with their parents at T1, controlled for their personal education (HR_not living with parents in 91-model2_ = 1.67 [95% CI 1.15–2.42]). HRs of personal education are especially pronounced for other violent deaths (excluding suicides and road accidents) and other natural diseases (excluding cancers and alcohol-related deaths), e.g. there is a more than tenfold risk of dying from other violent deaths among the primary educated compared to the highest educated, controlled for parental education (HR_personal PE-model2_ = 11.0 [6.7–18.1]).

Among women, similar results are found, with two important remarks. First, the relative inequalities in personal education are somewhat smaller than among men, albeit still being pronounced for each educational level compared to the highest educated and for most causes of death. No significant differences were found for cancers in model 1, while PE women have a twofold mortality rate compared to the HE (HR_PE-model2_ = 2.05 [1.03–4.09]). For other diseases exclusive of cancers, extremely high hazard ratios are found among the primary educated (HR_PE-model2_ = 20.2 [13.0–31.5]). Second, stronger associations are found between parental education and suicides and other violent deaths than among men. Significantly lower suicide mortality is found among women from PE families compared to women from HE families. For other violent deaths, having a deprived family background increases the mortality rates compared to women from HE families, after controlling for personal educational level (e.g. HR_PE-model2_ = 2.31 [1.05–5.06]).

### Adjustment for other SEP measures and longstanding illness

Finally, we look into the association between educational mobility and mortality hold after adjusting for other SEP measures (i.e. housing and employment situation) both in childhood and in young adulthood, and for having a longstanding illness at T2.

An instant answer to this question is presented in Figs 1 (men) and 2 (women), as a visualisation of the HR of each cross-classification of parental and personal education, with stable HE persons as the reference. While model 0 includes all 16 combinations of parental and personal education on all-cause mortality, the full model includes all control variables. Among men, the differences between higher educated persons and all other combinations of parental and personal education remain significant in the full model. The differences between primary, lower and higher secondary educated persons become less pronounced. Among women (Fig 2) the pattern is slightly different. More substantial differences are found for all downward mobile persons towards primary education (HR>7.0), while the stable PE persons have similar mortality rates as the stable HE in the full model, especially after controlling for having a longstanding illness at T2. In S1 and S2 Appendix, a stepwise inclusion of all significantly contributing factors is shown, with formal tests for interaction between personal and parental education.

## Discussion & Conclusion

### General findings

In answer to our first research question, the results showed that it is personal rather than parental education that is associated with inequalities in young-adult mortality. Although there were some small differences in young-adult mortality according to parental educational level, most of these differences disappeared after controlling for the personal education of the young adults. In other words, there is no confirmation of resource multiplication: the destination position seems to play the most important part in mortality risks. This was found for men and women, and for most causes of death. In line with Remes et al. [7], this study also showed that SEP characteristics of the parents are mainly indirectly associated with mortality in young adulthood, rather than directly. In spite of the trend towards upward mobility, there is still a higher share of young adults from high-educated families who are higher educated themselves compared to the share from low-educated families. Through the provision of the resources necessary for the educational success of their children, highly educated parents can thus indirectly influence the young-adult mortality risk of their children.

On the positive side, early childhood disadvantage could possibly be overcome with personal education, and its related job opportunities, providing support for the resource substitution theory [16]. Life course perspectives showed that health continuously develops over the life span, and an individual may move towards greater or lesser degrees of health due to several biological, social and environmental factors [47].

While there is little research focusing on intergenerational mobility and young-adult mortality, several studies were performed on subjective health and health-related behaviour in young adults [6,9,30,34–35]. Some found, as in our study, that the position of destination is most important [9,34–35], while others found persistent effects of childhood SEP [6,30]. Most of these persistent effects however probably operate indirectly through risk exposure and behaviours in adolescence. Hayward & Gorman [48] found that most associations between childhood socioeconomic and family circumstances on men’s mortality were largely indirect through own socioeconomic achievements and lifestyles. Several other studies found significant associations between parental social class and morbidity [49], all-cause and cause-specific mortality in older adult age [5,43,50] after controlling for personal social class or educational level.

### At-risk groups

Although personal education is associated more strongly with young-adult mortality, being downwardly mobile was associated with higher mortality rates than in stable low educated persons, a pattern found especially in women. In answer to our second research question, it was found that the mortality rate of the downwardly mobile equals the rate of the educational position of destination for most causes of death, similar to the relation found with all-cause mortality, irrespective of the educational origin, which is in line with other research [6]. All-cause mortality among women follows a divergent pattern: downwardly mobile young women have a higher mortality risk compared to the stable high-educated ones, while stable PE showed similar mortality risks as the high educated after controlling for having a longstanding illness at T2. This is in large part related to suicide mortality, for which other research also found higher suicide mortality among women from high-educated families [42,51]. As hypothesised by Strand & Kunst [42], high demands of high-educated parents could induce stress and lead to low self-esteem when not meeting the standards parents were hoping for, but so far, no research has confirmed this. This generates an interesting line of thought for further research.

If children between the age of 8 and 19 do not live with their parents, this clearly indicates a disturbed childhood. There can be several reasons for this alternative household position. In most cases, these young persons grew up with other family members (i.e. grandparents, siblings) because their parents could not provide the needed care or because both parents died. A small share might have lived in youth detention centres or with their partner. Being separated from your parents, and especially because of death, might lead to risk-taking behaviour, mental health problems and bad school performance due to loss of emotional and material support and structural changes in daily living [52], and may ultimately result in an increased mortality risk [53], especially for suicide and other external deaths, as found in our study. To what extent this disturbed youth hampers the educational path is still a matter of debate, but our results clearly indicate that being highly educated does not shield these young adults completely against the circumstances in which they grew up.

### Methodological considerations

Several studies on intergenerational mobility only collected information at one point in time, meaning that information on childhood characteristics is based on recall information of young adults. Such information is often less precise and more susceptible to measurement error than when measurements are taken at several points in time [6]. The data at hand do not have these limitations, as both parental and personal SEP factors are measured at two time points (at the time of census data collection), with an individual link between the information of the parents and the children. The other qualities of the database consist of its linkage with the population register and with the death certificates for the period 2001–2009, enabling a follow-up of the population for emigration and death and providing information on the causes of death.

Around one in five of the observed young adults were still enrolled in education at T2. Therefore we made use of their current educational level rather than their highest completed level. Sensitivity analyses showed that, when using only completed education, there is a significant underestimation of upward educational mobility (from HSE to HE) and there is an overestimation of young adults with the same educational level as their parents. As indicated in the methods section, some HR estimates changed when using only completed educational level, but the overall interpretations did not, and the conclusions were therefore similar.

Missing values exist for all variables included in the described models, and thus also for our key variables concerning parental and personal education. Less than 10% of the information was missing when combining information of parental and personal education. Cox regression was run including missing information on education as a separate category, but also excluding this information, leading to similar results. We need to take into account that this could lead to a small bias in the estimation of educational mobility, and that there may be some residual confounding in the Cox regression.

As young adults are a mobile group, both in terms of moving and employment, measures of housing status and activity status may have changed since the last measurement at T2 (the 2001 census). This cannot be controlled for in our models. However, home ownership probably reflects current as well as future wealth and wealth of the family of origin given the relatively young age of measurement, similar to the data design of Kuh [50].

Register-based population data normally do not include detailed information on health-related issues. The 2001 census is an exceptionally rich source of information in this respect, including questions on self-rated health and long-term illness and disability. With the data at hand, it is not possible to identify causal relations, but we found strong associations between having a longstanding illness and young-adult mortality, that clearly lowered the association between personal education and young-adult mortality, especially among the primary educated. It is possible that sickness kept them from attaining a higher educational level. But even among this low-educated group, the association with mortality remains significant after inclusion of having a long-standing illness, except for cancer mortality and suicide mortality among women. To account for health selection, we would also need information on health at T1 (the 1991 census) and longitudinal follow-up on health, which was not available.

### Policy implications & further research

This research showed that educational level is an important differentiator in young-adult mortality inequalities. It is one of the important upstream factors and our research results suggest that it represents an interesting path for improving health and reducing inequalities, as other authors also stress [24,54]. The long-term effects of educational investments cannot be denied; further improvements in educational attainment are still possible and can lead to substantial health gains [55]. Offering high-quality education for all should always be a top-priority for the Belgian government, or for any government in that respect. The Belgian educational system is characterised as being of high quality, but less efficient and equal than that of its neighbouring countries due to social origin still largely determining educational success [56]. Discussion is needed on how to restructure the educational system and how to reassess academic and vocational tracks, in order to mitigate fatigue and stimulate everyone’s own abilities.

## Supporting Information

S1 Appendix (DOCX)

S2 Appendix (DOCX)
