# Supplementary material for: Educational Inequalities in the Transition to Adulthood in Belgium: The Impact of Intergenerational Mobility on Young-Adult Mortality in 2001-2009
Source: PLoS One. 2015 Dec 14;10(12):e0142104. doi: 10.1371/journal.pone.0142104 (PMC4697474; doi:10.1371/journal.pone.0142104)
Supplement: S1 Appendix — (DOCX) [file pone.0142104.s001.docx]

**S1 Appendix: Hazard Ratios (HR) of all-cause mortality between 2001-2009, all models, men**

Study population: born between 1972 and 1982 and living in Flanders or the Brussels-Capital Region on 01/10/2001

*: p<0.050; **: p<0.010; ***: p<0.001

PE: primary education, LSE= lower secondary education, HSE=higher secondary education, HE=higher education
